# Supplementary material for: Unforeseen crystal forms of the natural osmolyte floridoside
Source: Commun Chem. 2020 Sep 11;3:128. doi: 10.1038/s42004-020-00376-z (PMC9814874; doi:10.1038/s42004-020-00376-z)
Supplement: Supplementary file 1 — Supplementary Information [file 42004_2020_376_MOESM1_ESM.pdf]

Supplementary Information: Unheralded forms of the natural osmolyte floridoside

Andrew J. Maneffa,[1] Adrian Whitwood,[2] A. Steve Whitehouse,[3] Hugh Powell,[3] James H. Clark,[1] Avtar S. Matharu,[1]*

[1]Green Chemistry Centre of Excellence, Department of Chemistry, University of York, Heslington, York YO10 5DD, United Kingdom (*email: avtar.matharu@york.ac.uk)

[2]Department of Chemistry, University of York, Heslington, York YO10 5DD, United Kingdom

[2]Nestlé Product Technology Centre (Nestec York Ltd.), Clifton, York, YO31 8FY, United Kingdom

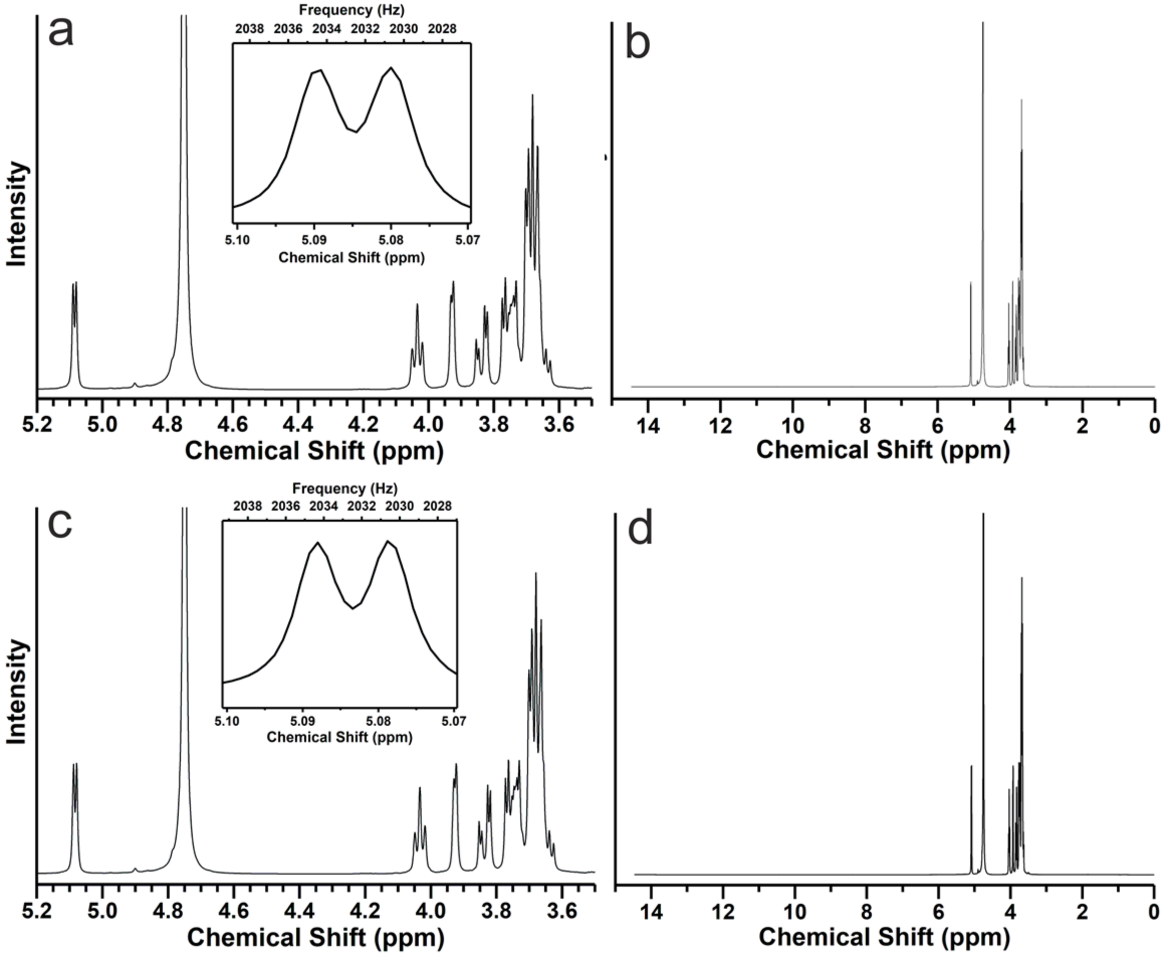

Supplementary Figure 1: ^{1}H NMR (400 MHz) of filtered UCM (**a** and **b**) and UCM-T (**c** and **d**) (Referenced with respect to HOD at 4.75 ppm).

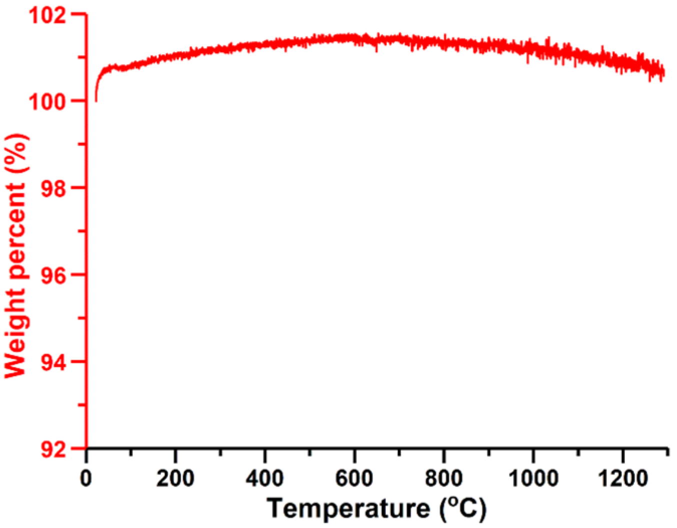

Supplementary Figure 2: Blank TGA run (100:20 mL min^{-1}, Air:N_2, 5 K min^{-1}) based on a 50 mg sample mass.

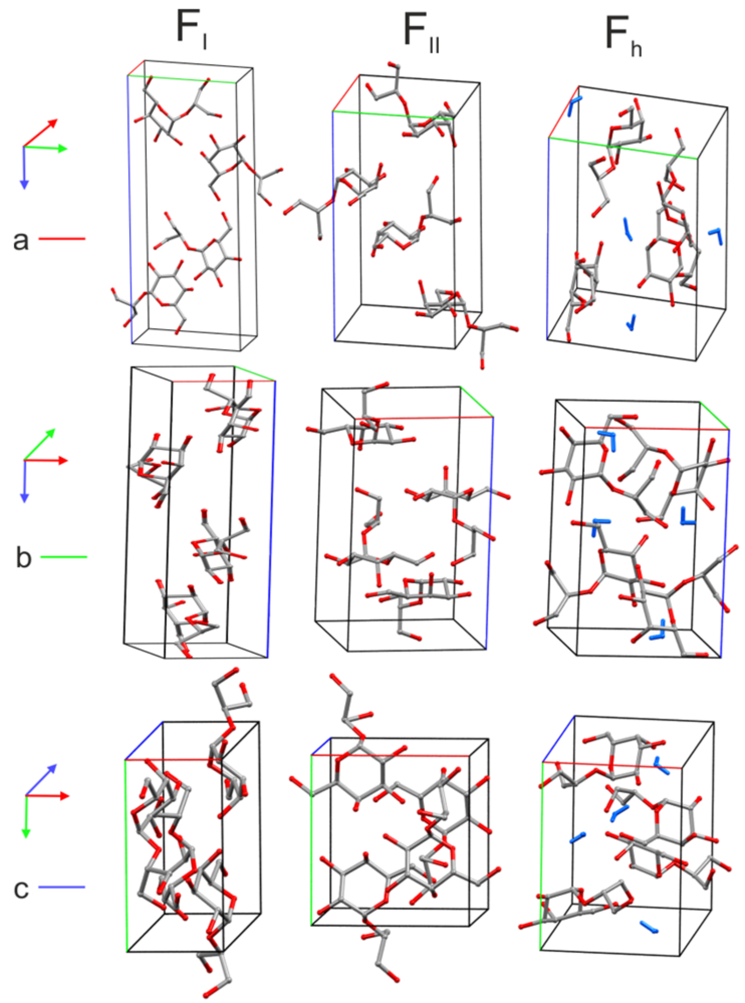

Supplementary Figure 3: Unit cells of (from 1st to 3rd column F_I, F_{II} and F_h viewed along the (from top to bottom) a-, b- or c axes (red, green or blue cuboid edges). Hydrogen atoms, except those belonging to H_2O (coloured blue) in F_h are omitted for clarity.

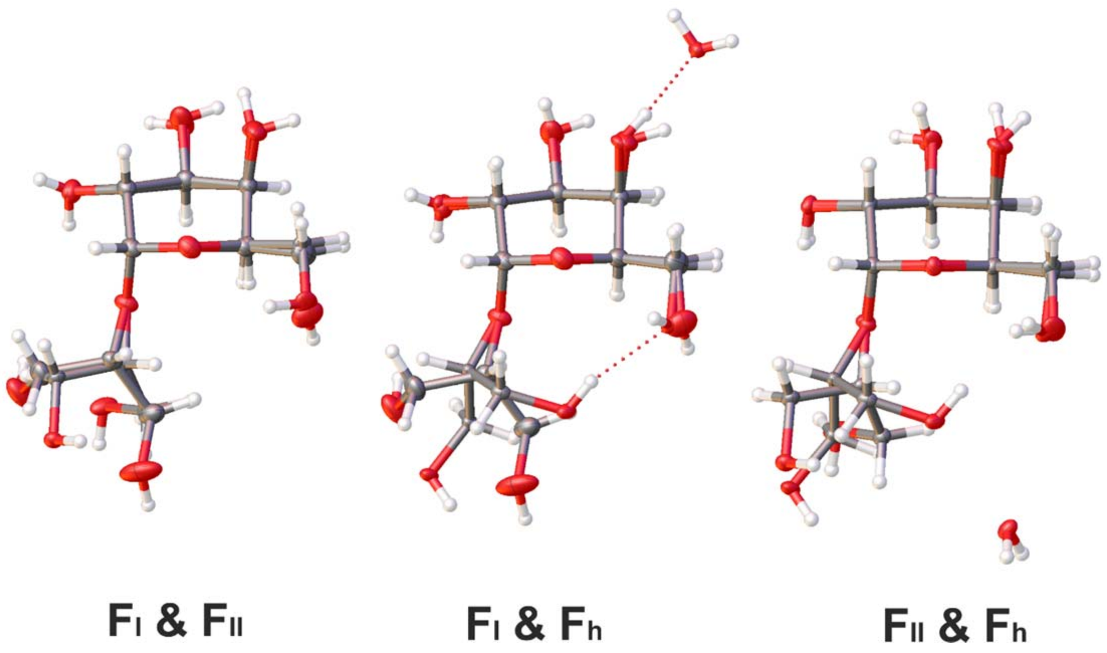

Supplementary Figure 4: Structural overlays of thermal ellipsoid representations for (from left to right) F_I & F_{II}, F_I & F_h, and F_{II} and F_h at the 50 % probability level wherein hydrogens are drawn with arbitrary radii.

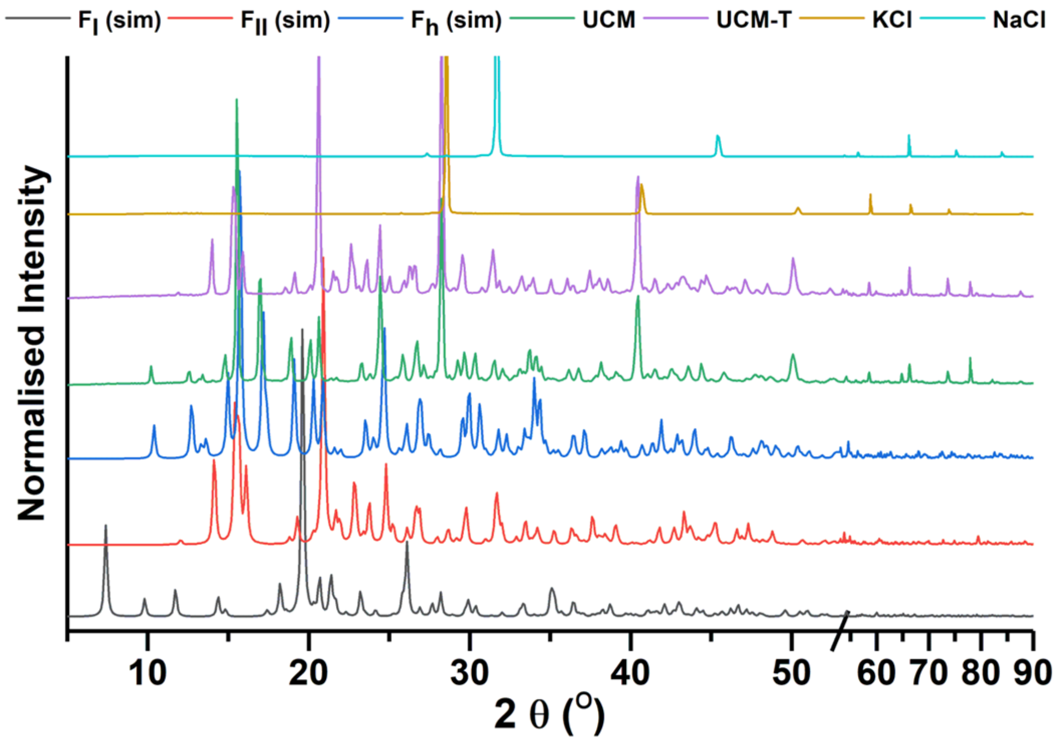

Supplementary Figure 5: Simulated pXRD traces (full width at half-maximum is 0.20) for F_I, FI_I and F_h with experimental UCM, UCM-T, KCl and NaCl spectra added for comparison.

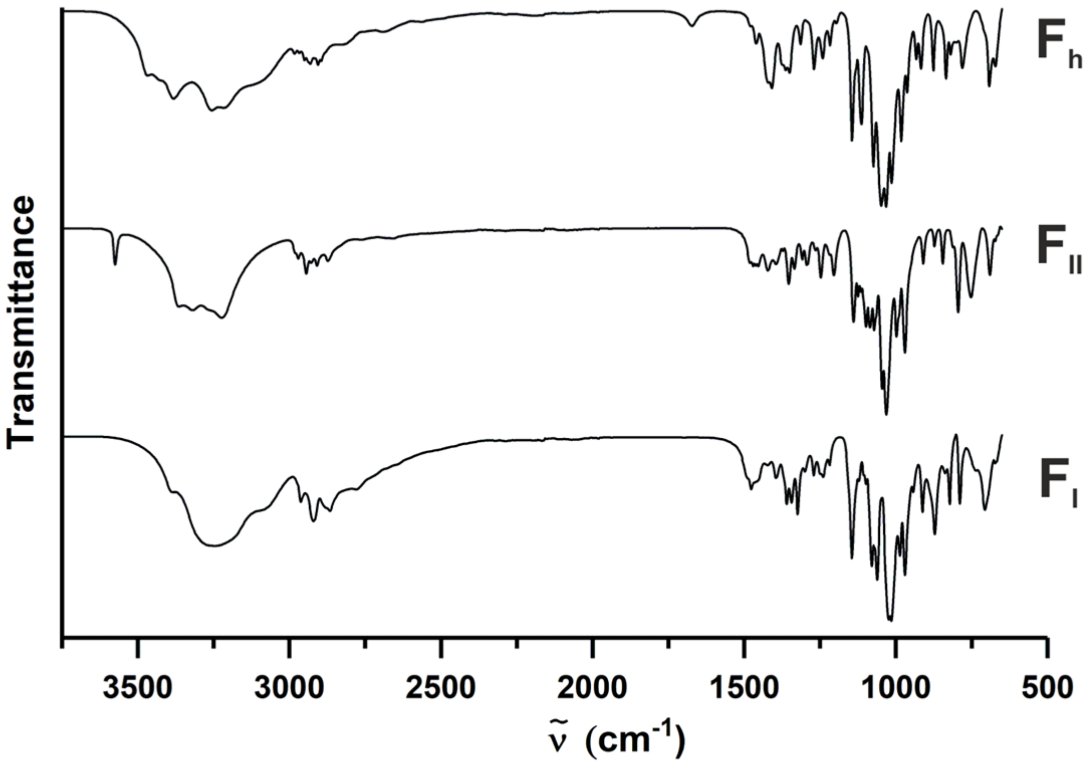

Supplementary Figure 6: ATR-FTIR spectra (650 – 3750 cm^{-1}) of pure crystalline F_h, F_{II} and F_I.

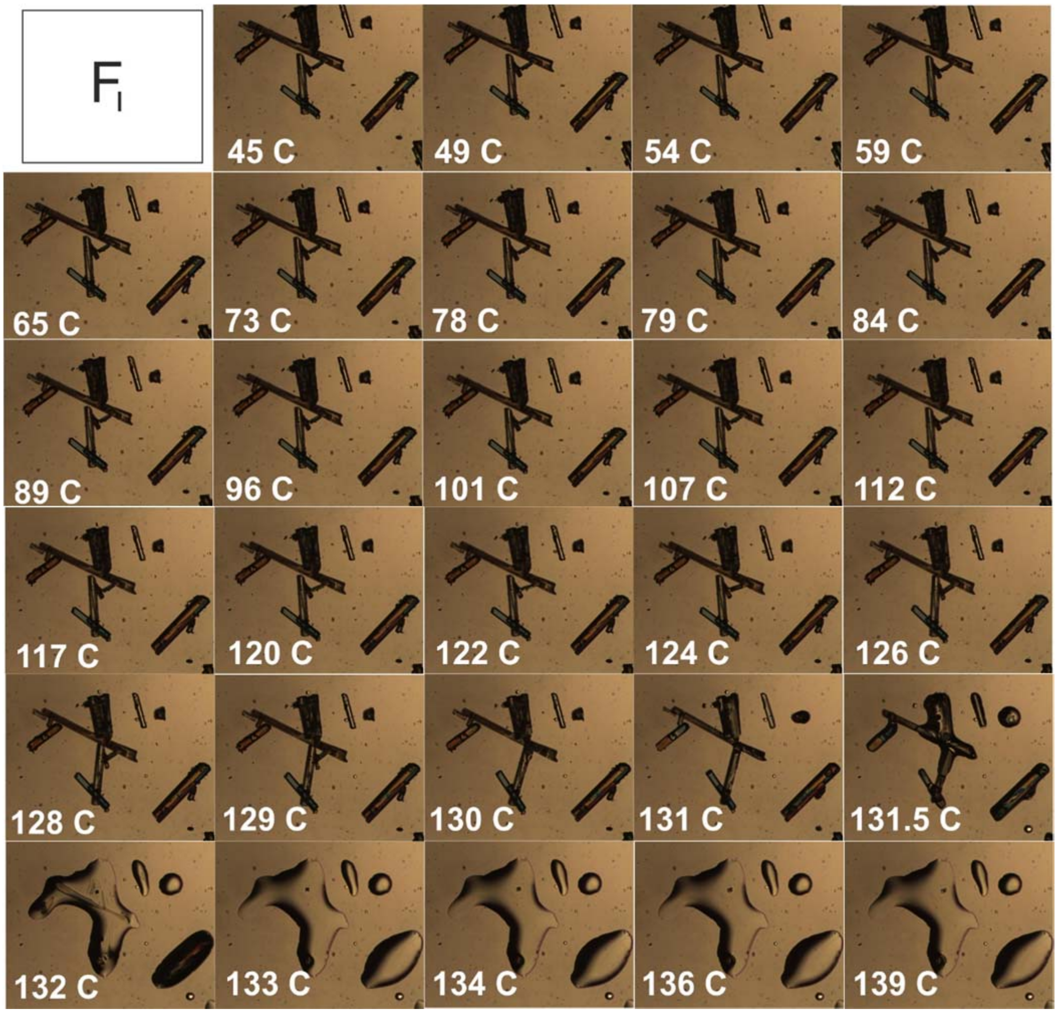

Supplementary Fig. 7: Hot-stage micrographs of crystalline F_I recorded upon heating from 45 to 139 ^oC.

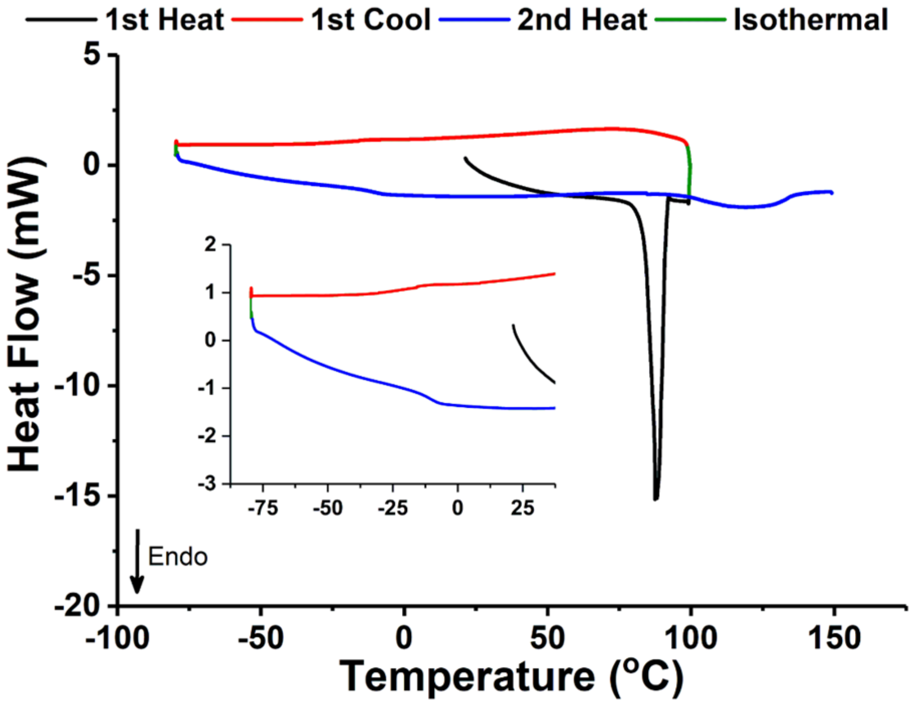

Supplementary Figure 8: DSC thermogram showing the; 1st heating (to 100 ^{o}C), 1st cooling (to -80 ^{o}C, 2nd heating (to 150 ^{o}C) and isothermal cycles for crystalline F_h (mg) at 5 K min^{-1}.

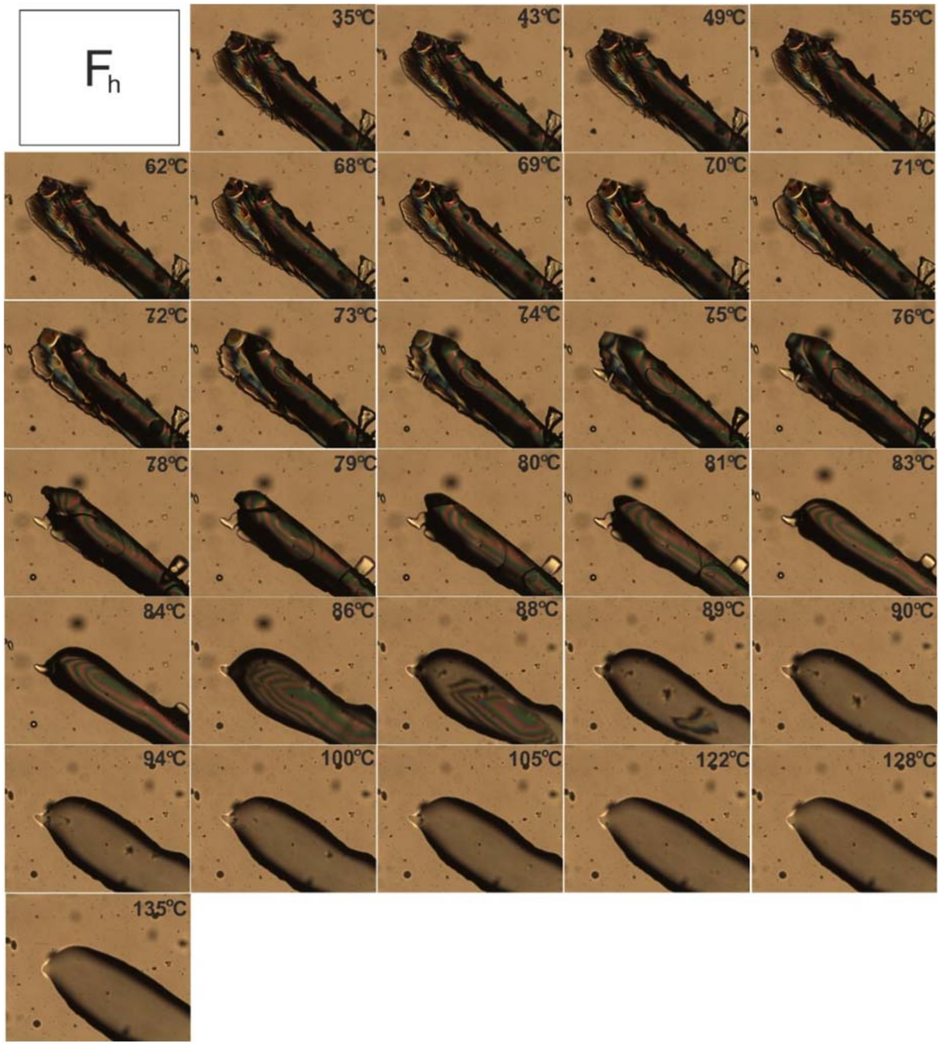

Supplementary Figure 9: Hot-stage micrographs of a single F_h crystal heated from 35 to 135 ^oC at a rate of 5 K min^{-1}.

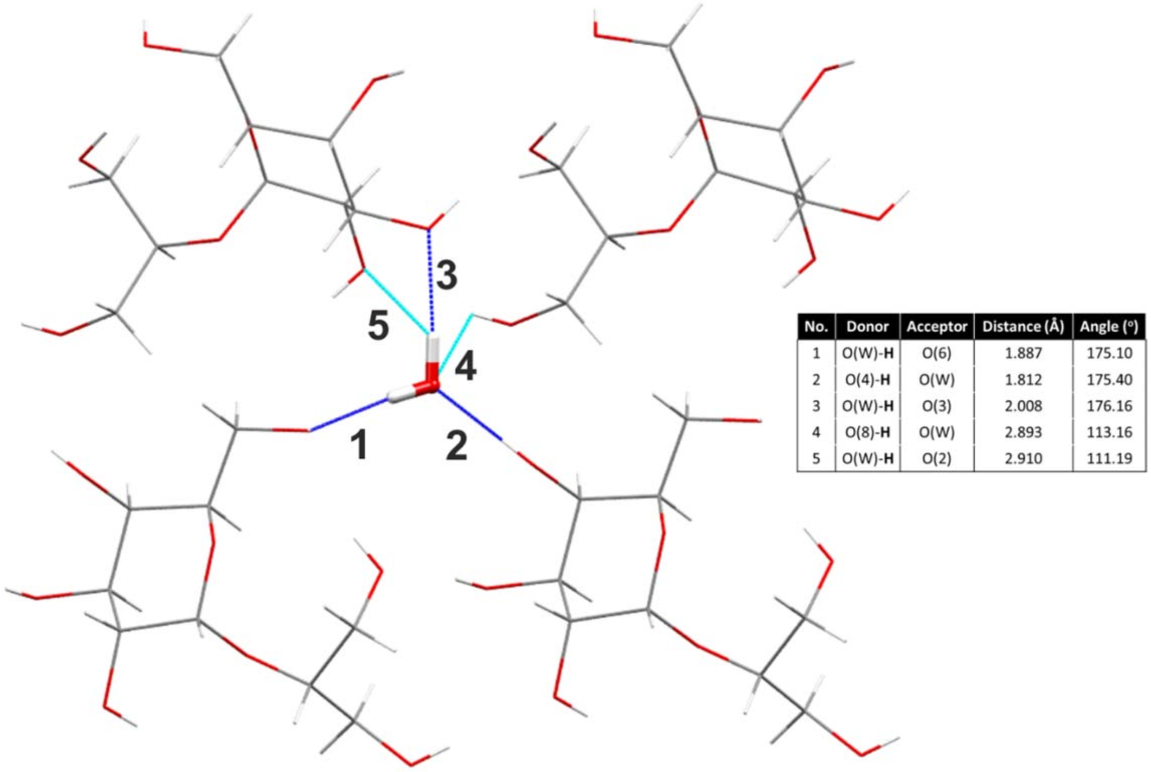

No.	Donor	Acceptor	Distance (Å)	Angle (^{o})
1	O(W)-**H**	O(6)	1.887	175.10
2	O(4)-**H**	O(W)	1.812	175.40
3	O(W)-**H**	O(3)	2.008	176.16
4	O(8)-**H**	O(W)	2.893	113.16
5	O(W)-**H**	O(2)	2.910	111.19

Supplementary Figure 10: Graphical and tabulated representation of hydrogen bonds found for the water (ball and stick) with the surrounding floridoside molecules (wireframe) in F_h as defined by the following criteria: < 3.00 Å (H···A) and > 90 ^{o} (O-H···A), OH donor only, and > 1 bond separation between intramolecular donor/acceptor. The navy or cyan dashed lines indicate whether a H···A length is ≤ 2.20 or 2.21 – 3.00 Å respectively and the bracketed numbers in the table denote the identity of the floridoside oxygen atom involved (w = water).

Supplementary Table 1: Tabulated crystal and structural refinement data for F_h (this work, CCDC 2004260), F_{II} (this work, CCDC 2004259) and F_I (Vonthron-Senecheau *et al.*, CCDC 680442).[1]

**Polymorph Code**	F_h	F_{II}	F_I
**Empirical formula**	$C_9H_{20}O_9$	$C_9H_{18}O_8$	$C_9H_{18}O_8$
**Formula weight**	272.25	254.23	254.23
**Temperature/K**	110.00(10)	110.00(10)	150
**Crystal system**	orthorhombic	orthorhombic	orthorhombic
**Space group**	$P2_12_12_1$	$P2_12_12_1$	$P2_12_12_1$
**a/Å**	8.22038(16)	8.54811(10)	4.88440(10)
**b/Å**	11.2533(3)	9.19251(10)	9.7259(3)
**c/Å**	12.9852(2)	14.34851(17)	23.8754(6)
**α/°**	90	90	90
**β/°**	90	90	90
**γ/°**	90	90	90
**Volume/Å^3**	1201.22(4)	1127.49(2)	1134.21(5)
**Z**	4	4	4
**ρ_{calc}g/cm^3**	1.505	1.498	1.489
**μ/mm^{-1}**	1.185	1.154	0.132
**F(000)**	584.0	544.0	544.0
**Crystal size/mm^3**	0.236 × 0.114 × 0.025	0.275 × 0.153 × 0.136	0.649 × 0.431 × 0.351
**Radiation**	CuKα (λ = 1.54184)	CuKα (λ = 1.54184)	MoKa (λ = 0.71073 Å).
**2Θ range for data collection/°**	10.402 to 134.146	11.432 to 133.962	2.70 to 38.67
**Index ranges**	$-9 \leq h \leq 9$, $-13 \leq k \leq 13$, $-15 \leq l \leq 8$	$-10 \leq h \leq 10$, $-10 \leq k \leq 10$, $-17 \leq l \leq 16$	$-8 \leq h \leq 8$, $-16 \leq k \leq 16$, $-41 \leq l \leq 41$
**Reflections collected**	8062	10041	63701
**Independent reflections**	2155 [R_{int} = 0.0235, R_{sigma} = 0.0199]	2012 [Rint = 0.0222, Rsigma = 0.0151]	6386 [R_{int} = 0.0264]
**Data/restraints/parameters**	2155/0/196	2012/0/179	6386/0/226
**Goodness-of-fit on F^2**	1.044	1.036	1.095
**Final R indexes [I>=2σ (I)]**	R_1 = 0.0221, wR_2 = 0.0545	R1 = 0.0215, wR2 = 0.0550	R_1 = 0.0326, wR_2 = 0.0852
**Final R indexes [all data]**	R_1 = 0.0234, wR_2 = 0.0554	R1 = 0.0223, wR2 = 0.0556	R_1 = 0.0392, wR_2 = 0.0894
**Largest diff. peak/hole / e Å^{-3}**	0.18/-0.14	0.23/-0.16	0.362/-0.21
**Flack parameter**	-0.02(8)	-0.02(6)	0.1(4)

Supplementary References

1. Vonthron-Senecheau, C., Santos, J., Mussio, I. & Rusig, A. X-ray structure of floridoside isolated from the marine red algae Dilsea carnosa. *Carbohydrate Research* **343**, 2697-2698, doi:10.1016/j.carres.2008.06.017 (2008), CCDC 680442.
