# Supplementary material for: Unforeseen crystal forms of the natural osmolyte floridoside
Source: Commun Chem. 2020 Sep 11;3:128. doi: 10.1038/s42004-020-00376-z (PMC9814874; doi:10.1038/s42004-020-00376-z)
Supplement: Supplementary file 2 — Description of Additional Supplementary Files [file 42004_2020_376_MOESM2_ESM.pdf]

Description of Additional Supplementary Files

File Name: Supplementary Data 1
Description: Crystallographic information file for F_h.

File Name: Supplementary Data 2
Description: Crystallographic information file for F_{II}.
